# SDMap: a comprehensive database of spatial drug perturbation maps

**DOI:** 10.1093/nar/gkaf1046

**Published:** 2025-10-22

**Authors:** Feng Li, Zhe Chen, Yifang Zhang, Xinguo Ye, Wenli Fan, Anhui Kang, Xinyu Song, Liying Pei, Quan Qi, Wensong Liu, Zhengliang Zha, Yunpeng Zhang, Chunlong Zhang, Yanjun Xu

**Affiliations:** College of Bioinformatics Science and Technology, Harbin Medical University, 157 Baojian Road, Harbin 150081, China; College of Bioinformatics Science and Technology, Harbin Medical University, 157 Baojian Road, Harbin 150081, China; College of Bioinformatics Science and Technology, Harbin Medical University, 157 Baojian Road, Harbin 150081, China; College of Bioinformatics Science and Technology, Harbin Medical University, 157 Baojian Road, Harbin 150081, China; College of Bioinformatics Science and Technology, Harbin Medical University, 157 Baojian Road, Harbin 150081, China; College of Bioinformatics Science and Technology, Harbin Medical University, 157 Baojian Road, Harbin 150081, China; College of Bioinformatics Science and Technology, Harbin Medical University, 157 Baojian Road, Harbin 150081, China; College of Bioinformatics Science and Technology, Harbin Medical University, 157 Baojian Road, Harbin 150081, China; College of Bioinformatics Science and Technology, Harbin Medical University, 157 Baojian Road, Harbin 150081, China; College of Bioinformatics Science and Technology, Harbin Medical University, 157 Baojian Road, Harbin 150081, China; College of Bioinformatics Science and Technology, Harbin Medical University, 157 Baojian Road, Harbin 150081, China; College of Bioinformatics Science and Technology, Harbin Medical University, 157 Baojian Road, Harbin 150081, China; College of Bioinformatics Science and Technology, Harbin Medical University, 157 Baojian Road, Harbin 150081, China; College of Bioinformatics Science and Technology, Harbin Medical University, 157 Baojian Road, Harbin 150081, China

## Abstract

The arrangement patterns and spatial distribution features of cells within tissues are key regulators of drug actions and cellular responses; however, the specific spatial microenvironment that mediates drug actions and efficacy remains to be elucidated. Here, we introduce SDMap (http://bio-bigdata.hrbmu.edu.cn/SDMap/), a database to dissect the spatial distributions of drug-associated cells and spatial contexts that contribute to drug actions. SDMap establishes connections between 5 490 079 spatial locations (spots/cells) derived from 989 slices of 35 human tissues (including disease-state slices) and 538 419 instances across 33 149 drugs. It also offers 10 user-friendly tools for retrieving and analyzing: (i) associations between drug-associated instances and spatial spots/cells, niches, or cell types; (ii) the influence of spatial drug target activity on drug actions; (iii) drug-associated instances exhibiting highly variable spatial perturbation effects; (iv) the perturbation effects of drug dosage and duration on the spatial microenvironment; (v) spatial differentiation trajectories of drug-perturbed cells; (vi) the impact of spatial cellular functional activity on drug perturbation; (vii) spatial perturbation effect maps of drug combinations; and (viii) 3D maps of drug perturbation effects within spatial tissue contexts. SDMap is anticipated to yield critical insights into how spatial heterogeneity influences drug actions, and serve as a comprehensive database for identifying spatially resolved precision therapy targets and elucidating drug-associated mechanisms.

## Graphical Abstract

**Graphical Abstract:**
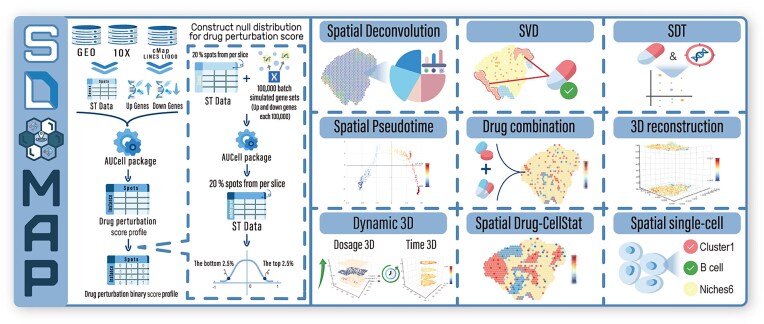

## Introduction

The composition, spatial arrangement, status, and interactions of cells within a tissue are highly heterogeneous, and this heterogeneity is precisely the key driving factor behind the differences in cellular drug responses and the diversity of therapeutic efficacy [1, 2]. Arora *et al.* integrated spatial transcriptomics (ST) data to reveal the structures of tumor cores and margins, which can predict survival rates and responses to targeted therapies [3]. Shiau *et al.* performed spatially resolved analyses of pancreatic cancer to uncover therapy-associated remodeling within the tumor microenvironment [4]. Therefore, in the context of spatial organization, determining the effects of drugs on cells and depicting the spatial distribution and heterogeneity of drug-perturbed cells within tissues are of crucial significance for revealing drug action mechanisms, promoting spatial cell-targeted therapy, and discovering new spatial therapeutic targets.

Advancements in ST technologies, which enable the profiling of gene expression dynamics in cells while retaining their inherent spatial contexts, have established themselves as a revolutionary approach for deciphering cellular spatial organizations and elucidating the underlying biological mechanisms [1, 5]. Recently, researchers have successfully identified cells or niches associated with phenotypes or traits in spatial organizations based on ST data. For example, Song *et al.* successfully integrated ST data to provide spatially resolved mapping of phenotype/trait-associated cells [1]. Zuo *et al.* proposed stClinic, which integrated spatial multi-omics data and phenotype data to discover clinically relevant niches based on dynamic graph model [2]. In addition, researchers have devised effective strategies to identify cells/cell subpopulations that respond to perturbations (e.g. drug treatment) from untreated single-cell datasets [6–9]. These studies enabled us to determine the effect of drug perturbation on cells within tissue space, and explore the distribution of cells affected by drug perturbations within spatially tissue slices, as well as the impact of spatial heterogeneity on the action of drugs. Currently, extensive ST and drug perturbation data resources have been generated. For example, databases such as STOmicsDB [10], SORC [11], and CROST [12] house manually curated ST datasets and also offer downstream analytical and visualization modules. SPathDB [13] is a database that integrated ST and biological pathway data to dissect the spatial heterogeneity of functional activity. The SODB [14] database archives datasets generated by diverse spatial omics technologies and incorporates interactive analysis functionalities. Furthermore, the CMap [15] and LINCS L1000 [16] data resources store a large amount of drug perturbation data. However, a comprehensive database that carefully curates these scattered valuable data resources to provide spatially resolved information of the perturbation effects of drugs on cells, to dissect the influence of spatial heterogeneity on cellular drug responses, drug efficacy, and the related mechanisms of drug action is still lacking.

Here, we developed SDMap (http://bio-bigdata.hrbmu.edu.cn/SDMap/), which intends to dissect the effects of drugs on cells within the spatial tissue and the spatial heterogeneity of drug action from multiple spatial dimensions. SDMap collected large-scale ST data and drug perturbation data and established the association between drug perturbation and spatial spots (cells). Currently, SDMap contains: (i) 5 490 079 spatial locations (consisting of 2 400 955 spot-level locations and 3 089 124 single-cell-level locations) from 989 slices of ST that corresponding to 35 tissue types; (ii) 538 419 treatment instances (i.e. 538 419 combinations of drug-dosage-duration time-cell context perturbation conditions) for 33 149 drugs; and (iii) associations among 5 490 079 spatial locations and 538 419 treatment instances for 33 149 drugs under different concentrations, treatment durations, and dosages. SDMap also contains associations among drug perturbation and spatial cluster, cell types, and niches, respectively. SDMap provides several flexible tools to facilitate retrieval, visualization, and analysis of data. For example, SDT (spatial drug target) tool provides the spatial distribution of drug target activities and its correlation with the perturbation effects of drug under different dosages and treatment durations; the Spatial Deconvolution and Spatial Drug-CellStat tools enable users to dissect the impact of spatial cellular identity and functional activity on drug perturbation; the Spatial Pseudotime tool offers the distribution of drug perturbation spots/cells along the spatial differentiation trajectory; the Drug combination tool enables to explore spatial perturbation effect map of drug combination under different concentrations, treatment durations and dosages; the 3D tool panel contains 3D reconstruction, Dosage 3D and Time 3D tools enable users to dissect three-dimensional distribution and dynamic changes of drug perturbation effects in spatial tissue. In summary, SDMap will provide important insights for investigating the influence of spatial heterogeneity on the action of drugs, and serve as a comprehensive database for discovery of spatial-dimensional precision therapy targets and dissecting drug-related mechanisms.

## Materials and methods

### Data collection and processing

#### Collection and processing of spatial transcriptome sequencing data

SDMap acquires spatial transcriptomic sequencing datasets from 10x Genomics (https://www.10xgenomics.com), GEO [17] (https://www.ncbi.nlm.nih.gov/geo/), and ST-related literature published in recent years. For each spatial transcriptomic dataset, detailed information such as patient ID, sample ID, tissue type, primary/metastatic lesion, and sequencing technology is integrated into SDMap. For the count expression matrices in these datasets, the SCTransform function from the R package Seurat [18] is used to normalize the expression data. SDMap has collected a total of 989 spatial transcriptomic slices across 35 tissue types, including 969 slices of spot-resolution spatial transcriptomic data and 20 slices of single-cell resolution spatial transcriptomic data, involving 5 490 079 spatial locations and cells (comprising 2 400 955 spatial spots and 3 089 124 spatial cells). SDMap employs the UMAP (Uniform Manifold Approximation and Projection) clustering algorithm, which is incorporated in the Seurat R package [18], to identify spot/cell clusters on each spatial slice. Principal component dimensionality reduction method is used to reduce the dimensionality of the normalized expression data. Principal component analysis (PCA) is performed on the normalized spatial spot expression matrix, and spatial spots are clustered using the Louvain algorithm with a resolution of 0.5. Unsupervised clustering is then conducted, where the KNN method is used to calculate neighbor distances, and spatial spots are iteratively clustered through modular optimization techniques. Finally, the UMAP algorithm is applied to project high-dimensional spatial spot data into a two-dimensional space, such that spatial spots with similar expression patterns are grouped into the same cluster.

#### Collection and processing of drug-related gene sets

SDMap collects drug perturbation-related genes from the CMap LINCS L1000 (level 5) dataset [16] (https://clue.io/data/). In the SDMap database, drugs, small molecules, and their stereoisomers from the LINCS L1000 dataset are collectively referred to as “drug”. A combination where a drug acts on a cell line (cell context) under a specific concentration and for a certain duration is defined as an “instance”. For each instance, a pair of perturbed upregulated and downregulated signature gene sets are constructed. Briefly, the identification of differentially expressed genes (upregulated/downregulated genes) in drug-related gene sets is directly based on the standardized data of LINCS L1000 dataset Level 5 publicly available from the CLUE database (https://clue.io/), with details accessible at https://clue.io/connectopedia/data_levels. The Level 5 dataset contains corrected *z*-score vectors, which are generated through a procedure that calculates differential expression values for genes by comparing each gene’s expression in the corresponding sample with respect to the entire plate population [16]. For each drug-related instance in the LINCS L1000 dataset [16], genes with a corrected *z*-score of ≥2 (or ≤−2) are identified as upregulated (or downregulated) differentially expressed genes in a specific signature (i.e. the signature gene sets of different instances). In addition, the compound information within the LINCS L1000 dataset, including details such as cmap name, target, and moa, was also obtained from the URL: https://clue.io/data/CMap2020#LINCS2020.

#### Collection and processing of scRNA-seq data

SDMap simultaneously collects scRNA-seq data corresponding to the tissue types of spot-resolution spatial tissue slices for cell type deconvolution (Supplementary Table S1). For the collected scRNA-seq datasets used for deconvolution, we employed the Seurat R package [18] to filter out genes expressed in <3 cells and exclude cells with <500 expressed genes. In particular, for scRNA-seq data paired with spatial transcriptomic data, cells expressing fewer than 200 genes were filtered out. We then merged samples by tissue type and normalized the expression profiles using the NormalizeData function. Dimensionality reduction and clustering were subsequently performed on the scRNA-seq data, and the FindAllMarkers function was used to identify marker genes for each cluster. We combined two automated methods, scMayoMap [19] and SingleR (https://doi.org/doi:10.18129/B9.bioc.SingleR), with manual annotation strategies to annotate cell types (Supplementary data). For cancer-related scRNA-seq datasets, the inferCNV method (https://github.com/broadinstitute/infercnv) was further used to identify malignant cell types (Supplementary data).

#### Acquisition of cancer-related functional gene sets and calculation of spatial activity scores

SDMap integrates three categories of cancer function-related gene sets, including: (i) 41 experimentally validated oncogenic pathways collected in our previous study (the related database URL is http://bio-bigdata.hrbmu.edu.cn/CPAD/) [20]; (ii) 35 Gene Ontology (GO) term gene sets classified as cancer hallmarks, downloaded from the GO database [21] with reference to the study by Hanahan *et al.* [22]; and (iii) 14 signature gene sets related to the functional status of cancer cells obtained from the CancerSEA database (http://biocc.hrbmu.edu.cn/CancerSEA/) [23]. SDMap calculates cancer-related functional activities at different spots on spatial tissue slices using the AUCell method [24].

#### Spatial deconvolution and cell annotation

For spot-resolution ST data, we used single-cell expression profiles of the same tissue type or cancer type (in the case of cancer tissue slices) as references. We removed cell types with fewer than 25 cells from the reference single-cell data, and subsequently performed deconvolution analysis on spatial spots using the run.RCTD function in the R packages spacexr [25] and STdeconvolve [26] to obtain the proportion distribution of different cell types within each spot on the slice. Further, based on the results of spatial spot deconvolution, to identify spots enriched with specific cell types, the cell type label with the highest proportion in a spot was assigned to the corresponding spot, facilitating the analysis of the distribution of different cell types on spatial tissue slices and the association between spatial cell identities and drug perturbations.

In addition, for single-cell resolution ST datasets, manual cell type annotation was performed based on cell marker genes obtained from the CellMarker 2.0 database [27].

#### Determination of spatial niches

SDMap identifies niches on spatial tissue slices using the BuildNicheAssay function from the Seurat R package [18]. By referring to the identification of 12 stable niches in the study by Vannan *et al.* [28], the BuildNicheAssay function is applied to construct 12 distinct niches for the target dataset (spatial tissue slice). This allows for a detailed presentation of the distribution of spatial spots across different niches, providing robust support for in-depth investigations into niche differentiation of spatial cell populations and their associations with biological functions.

#### Establishing connections among drug treatment instances and spatial spots/cells

SDMap calculates the perturbation effect score (*IS*) of drug-related specific instance on spatial spots based on the upregulated and downregulated signature gene sets associated with drug-related treatment instance (i.e. certain dosage, duration, and cell context), as well as the expression data of spots/cells on spatial slice. SDMap employs the AUCell method [24], which is specifically developed for single-cell data, to evaluate the activity of upregulated and downregulated signature gene sets of drug-related instances in spots or cells (for single-cell resolution ST data) on spatial slices, and establishes instance-spatial spot associations. When inferring the perturbation effect of an instance on a spatial spot, the relationship between the upregulated and downregulated genes perturbed by the drug-related instance and the ranked list of gene expressions within the spatial spot/cell is comprehensively considered. The calculation of the perturbation score *IS_ij_* of drug-related instance *i* (i.e. under the context of specific dosage, duration, and cell line of action of the drug) on a certain spatial spot *j* in the tissue slice is as follows:


\begin{eqnarray*}
I{{S}_{ij}} = {\rm AUC\;score}_{ij}^{up} - {\rm AUC\;score}_{ij}^{down}.
\end{eqnarray*}

Here, ${\rm AUC\;score}_{ij}^{up}$ (${\rm AUC\;score}_{ij}^{down}$) represents the AUC score value calculated for the upregulated (downregulated) signature gene set perturbed by instance *i* in spatial location *j*, respectively. AUCell leverages the “Area Under the Curve” (AUC) to determine whether a key subset of the input gene set is enriched at the top of the expression ranking for each spot/cell. Specifically, the AUC reflects both the proportion of expressed genes within the signature and their relative expression levels in comparison to other genes within the spot or cell [24]. Thus, if *IS_ij_* > 0, it indicates that the highly expressed genes in spatial spot *j* are more consistent with the genes upregulated by instance *i* perturbation compared to the gene set downregulated by instance *i* perturbation. In this case, when instance *i* acts on spatial spot *j*, its promoting (upregulating) effect on the naturally highly expressed genes in the spatial location is stronger than its inhibiting (downregulating) effect. That is, *IS_ij_* > 0 reflects that the instance *i* has a promoting effect on the original state of spatial spot *j*. Conversely, if *IS_ij_* < 0, it means that the highly expressed genes in spatial spot *j* are more consistent with the genes downregulated by instance *i* perturbation compared to the gene set upregulated by instance *i* perturbation. Thus, when instance *i* acts on spatial location *j*, its inhibiting (downregulating) effect on the naturally highly expressed genes in the spatial spot is stronger than its promoting (upregulating) effect. In other words, *IS_ij_* < 0 reflects that the instance has an inhibiting effect on the original state of spatial spot *j*. SDMap calculates the perturbation effect scores between drug-related instances and spatial locations, and for each spatial tissue slice, constructs an instance-spatial spot perturbation effect scoring matrix. Here, it is ensured that the cell line environment in which the instances included in the matrix act is consistent with the tissue type to which the spatial slice belongs.

Further, the significance of the perturbation effect of drug-related instances on spatial spots was evaluated. SDMap constructs a null distribution of the perturbation effect score (*IS*) of drug-related instances on spatial spots (Supplementary data). Subsequently, a significance threshold is determined based on the constructed null distribution of *IS* scores: all background scores are sorted, and the scores corresponding to the top 2.5% (bottom 2.5%) of the sorted results are taken as the thresholds for significant promotion (significant inhibition) of spatial spots by drug-related instances. Finally, a binary matrix of perturbation effects between drug-related instances and spatial spots is constructed according to the determined thresholds. For values in the instance-spatial spot perturbation effect *IS* scoring matrix that are greater than (less than) the significant promotion (inhibition) threshold, they are defined as the instance having a significant promotion (inhibition) effect on the corresponding spatial spot, and assigned a value of 1 (−1); the remaining values in the matrix are assigned a value of 0, indicating that the corresponding instance has no significant perturbation effect on the spatial spot.

#### Identifying drug treatment instances associated with spatial niches

For different slices, SDMap uses the hypergeometric test to evaluate whether the spots/cells promoted (inhibited) by drug treatment instances are significantly enriched in different niches of spatial tissue slices, thereby identifying the spatial niches promoted (inhibited) by drug treatment instances. The process for determining the effect of drug treatment instance *i* on spatial niche *s* is as follows: (i) The hypergeometric test was used to separately calculate the significance *P*-values for the enrichment of spatial spots/cells promoted and inhibited by instance *i* perturbation in niche *s*, and the *P*-values are adjusted using the “fdr” method. *P*_1_ (*P*_2_) represents adjusted *P*-value of enrichment of spots/cells that are promoted (inhibited) by instance *i* in niche *s*; (ii) If *P*_1_ < .05, *P*_2_ ≥ .05, and the ratio of the number of spots promoted by instance *i* to the number of spots inhibited by instance *i* in niche *s* is greater than 2, then instance *i* is considered to have a promoting effect on niche *s*; (iii) If *P*_2_ < .05, *P*_1_ ≥ .05, and the ratio of the number of spots inhibited by instance *i* to the number of spots promoted by instance *i* in niche *s* is greater than 2, then instance *i* is considered to have an inhibiting effect on niche *s*; and (iv) Otherwise, instance *i* is considered to have no significant association with niche s. In addition, SDMap uses a similar strategy to identify spatial cell types related to drug treatment instances.

#### Determination of the combined drug perturbation effects on spatial spots

SDMap integrates the perturbation effect scores of two instances to determine the combined effect of drug pairs on spatial spots. First, for each spatial spot, the mean value of the perturbation scores of the two instances is calculated as the combined perturbation effect of the drug pair on that spatial spot, which comprehensively reflects the perturbation intensity of the combined action of the two instances on the spatial spot. Then, the combined perturbation effects of drug pairs on spatial spots are categorized into three types: (i) synergistic promotion, where the perturbation effect scores (*IS*) of both instances on the spatial spot are greater than 0; (ii) synergistic inhibition, where the perturbation effect scores (*IS*) of both instances on the spatial spot are less than 0; and (iii) antagonism, where one of the perturbation effect scores (*IS*) of the two instances on the spatial spot is greater than 0 and the other is less than 0.

#### Determination of spatial highly variable instances

To calculate the degree of deviation between the perturbation consistency of an instance within spatial neighborhoods and the global perturbation pattern, SDMap identifies instances with significant spatial variation in their perturbation effects, providing key clues for dissecting the spatial heterogeneity of instance perturbations. First, referring to previous research [13], the perturbation effect scores (*IS*) are subjected to non-negative transformation (for details, see Supplementary data), and the FindSpatiallyVariableFeatures function in the Seurat R package [18] is used to evaluate the variation degree of perturbation effects of different instances across spatial spots. Then, based on the obtained statistical results, the identified spatially highly variable instances are ranked according to Moran’s I. SDMap selects the top 50 instances with the highest Moran’s I values as spatially highly variable instances.

#### Construction of spatial pseudotime

SDMap employs Monocle [29] to calculate the pseudotime series of spots on spatial slices and construct pseudotime trajectories. As a commonly used analytical tool for single-cell and ST data, this package can integrate gene expression characteristics with spatial location information to explore underlying patterns in the data, such as cell state transitions and dynamic changes in gene expression.

#### Reconstruction of 3D ST data

For ST data from multiple tissue slices of the same tissue in the same patient, SDMap performs spatial domain identification and multi-slice alignment using the Splane and Scube models from the SPACEL package [30]. Specifically, the Splane model is first initialized, and after training, it identifies spatial domains for each sample. Then, the Scube.align function is used to spatially align multiple slices based on the identified spatial domain labels, achieving positional matching of different slices in the 2D plane. After alignment, the *Z*-axis spacing between slices is calculated (determined based on the average distance of cells within the sample), and *Z*-axis information is added to the 2D coordinates of each slice to expand them into 3D coordinates. Subsequently, a point cloud is constructed using Open3D (https://www.open3d.org/), and a 3D mesh of the tissue is generated by combining the create_mesh function from SPACEL.Scube.utils_3d. Finally, sampling is performed inside and on the surface of the mesh using sample_in_mesh to obtain predicted 3D coordinate points.

#### Database construction

SDMap can be accessed free of charge via the URL http://bio-bigdata.hrbmu.edu.cn/SDMap/. This online web server was built on a Linux server utilizing Java Server Pages within the Tomcat software (version 6). For the documentation and management of datasets, the MySQL data server (version 5.6) was employed. A variety of JavaScript packages, such as jQuery, Datatables, and ECharts, were incorporated to facilitate the generation of result data and the realization of multilevel data visualization. All data processing and statistical analyses were conducted using R software (version 4.1.0). The SDMap website is compatible with commonly used web browsers like Google Chrome, Microsoft Edge, Firefox, and Safari, and it requires no registration or login procedures.

## Results

### Data collection and content of SDMap

**Figure 1.**
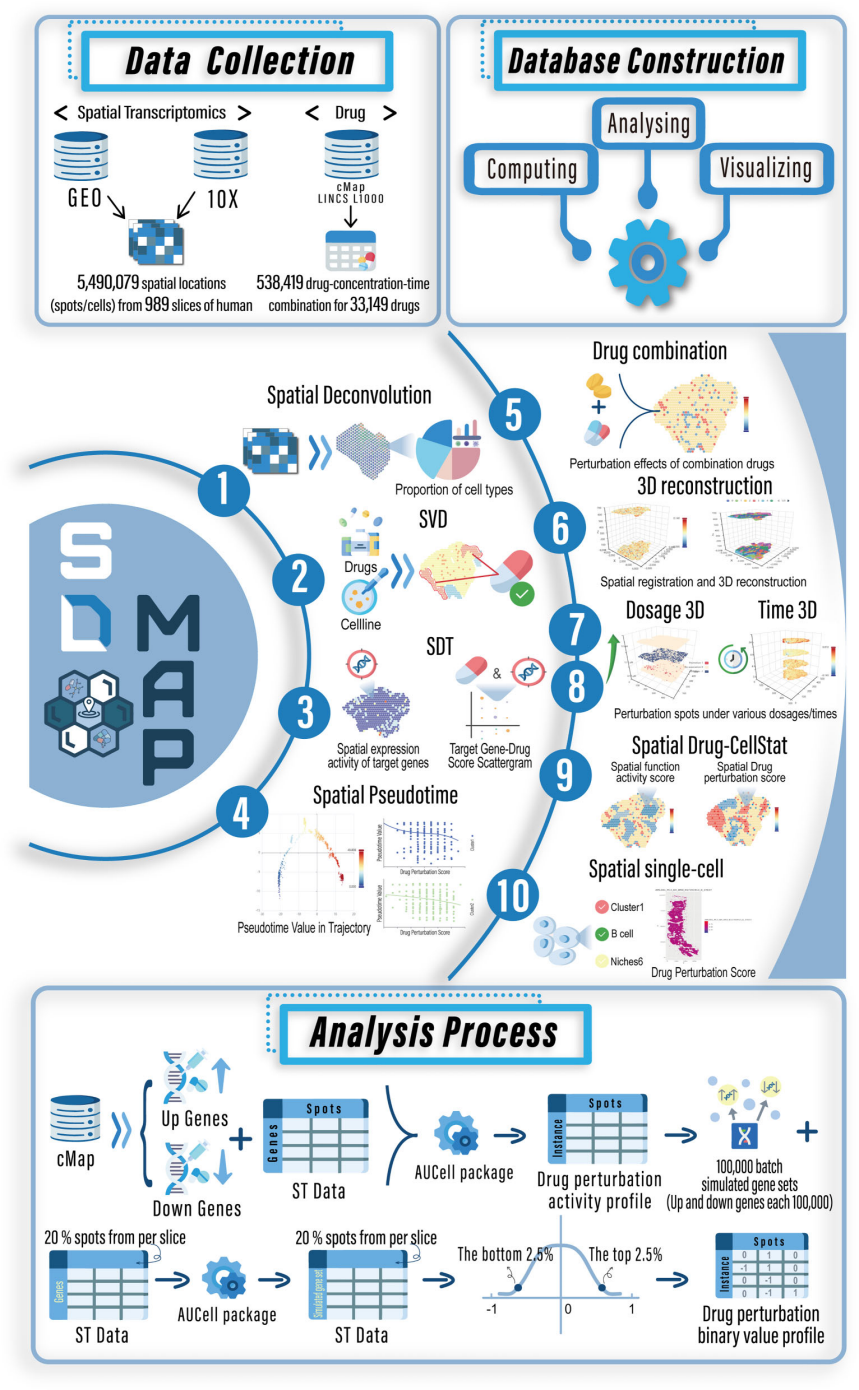
Overview of data content and user interface in SDMap. The upper left area shows data collection in SDMap, including spatial transcriptome datasets and drug perturbation upregulated and downregulated genes. The upper right area shows the construction process of SDMap. The middle area contains user interfaces to retrieve, analyze, and visualize data. The bottom area illustrates the identification of drug-spatial location associations (connections).

The present version of SDMap encompasses 5 490 079 spatial locations (comprising 2 400 955 spot-level and 3 089 124 single-cell-level locations) derived from 989 ST slices of 35 tissue types, 538 419 treatment instances for 33 149 pharmacologic agents (drugs), and multidimensional associations linking the 5 490 079 spatial locations with 538 419 treatment instances of drugs. Notably, SDMap further catalogs relationships between drug perturbations and spatial niches/cell types. An overview of SDMap is shown in Fig. 1.

SDMap has collected spatial transcriptome slices generated by various sequencing technologies (such as 10x Visium and Xenium). These include 969 spot-resolution and 20 single-cell-resolution slices derived from 121 datasets, corresponding to 35 tissue types. These 989 slices total contain 5 490 079 spatial spots/cells. The clinical information for each slice was also collected, including tissue, disease state, etc. There were 667 slices curated from 91 human diseases/disease subtypes, such as cancer. After data preprocessing, spot-resolution tissue slices contain an average of 2478 spatial spots per slice, while cell-resolution slices contain an average of 154 456 cellular locations per slice. For each slice, SDMap performs cell type deconvolution or cell annotation, cell clustering, and niche identification. SDMap incorporated the upregulated and downregulated signature gene sets corresponding to 538 419 treatment instances, specifically, 538 419 unique combinations of drug-concentration-duration time-cell context perturbation conditions for 33 149 drugs, which are derived from the LINCS L1000 dataset [16]. Within the upregulated and downregulated signature gene sets for 538 419 treatment instances, a total of 12 319 genes are involved. The statistical information of ST and drug perturbation data collected by SDMap can be found on the “Statistic” page of the SDMap database.

For each slice, SDMap employs the AUCell method [24] to assess the activity of signature gene sets for drug-induced perturbation at spatial spot/cell locations under specific treatment instance. This establishes multi-dimensional associations that link 5 490 079 spatial locations with 538 419 treatment instances (see the “Materials and methods” section) (Fig. 1). Additionally, SDMap also catalogs relationships between drug treatment instances and spatial niches/cell types.

To investigate the spatial heterogeneity of drug action and identify key driving features, SDMap characterizes drug actions underlying the cell types, clusters, and niches in the context of the spatial microenvironment. SDMap also constructs spatial developmental trajectories for each tissue slice and performs three-dimensional reconstruction of spatial slices. Furthermore, SDMap integrates external features to dissect drug actions in spatial microenvironment. These features include: (i) the SMILES structures and target information of drugs; (ii) cancer hallmarks, specifically gene sets of GO terms [21] that map to the hallmarks of cancer [31]; (iii) the cellular states of cancer cells [23]; and (iv) cancer-associated oncogenic pathways derived from CPAD [20]. SDMap database offers flexible access, analysis, and visualization of the aforementioned data, providing crucial data and tool support for investigating the spatial heterogeneity of drug actions, uncovering drug mechanisms, and discovering spatial therapeutic targets (Fig. 1).

### Features and utilities of SDMap

#### User-friendly search and browse interface for data inquiry and accession

**Figure 2.**
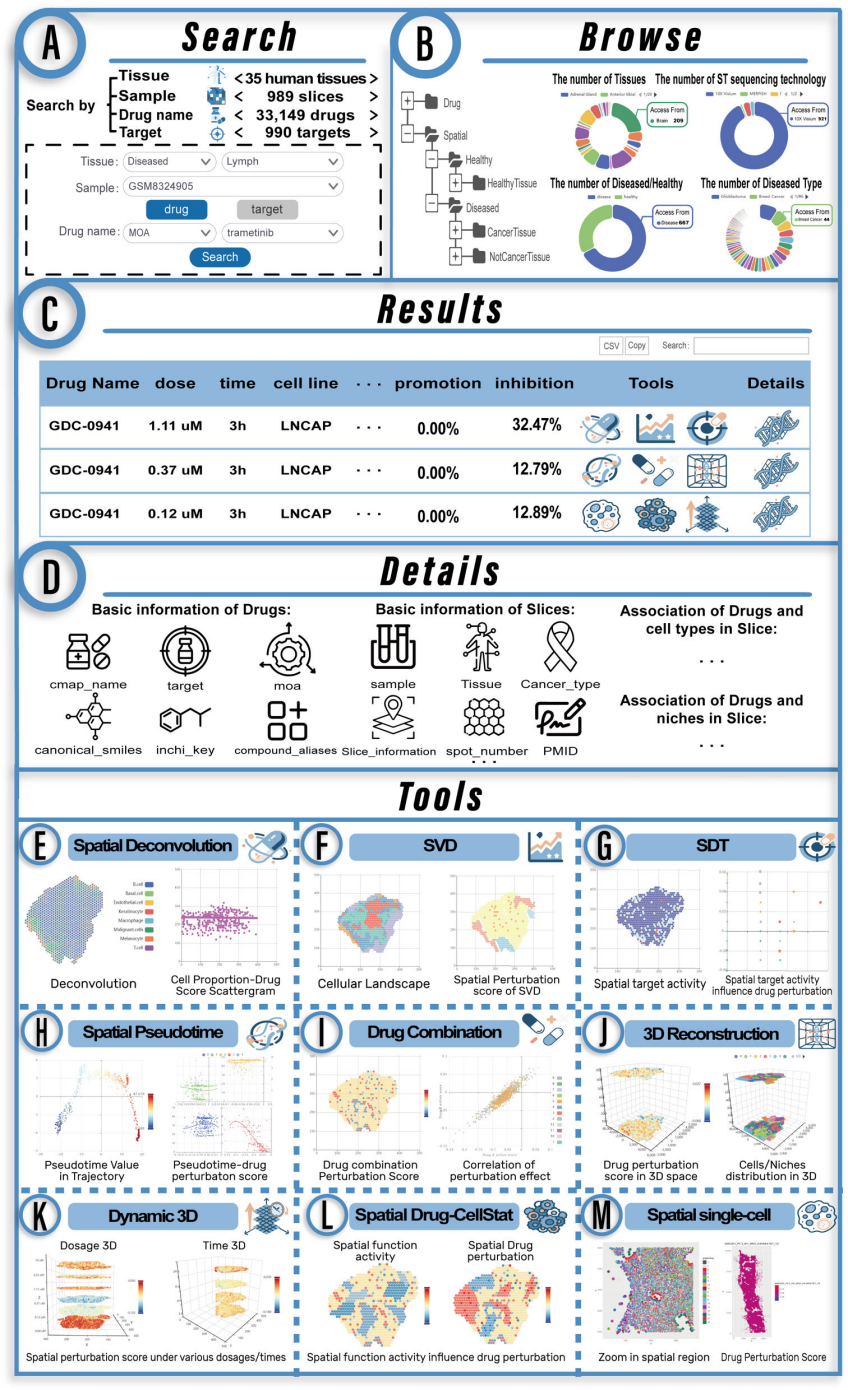
Features and utilities of SDMap. (**A**, **B**) The search and browse interfaces of SDMap. (**C**) The result table of search and browse of SDMap. (**D**) The detailed description of associations between drug and spatial locations. (**E**–**M**) Panel of flexible tools is used to explore the spatial heterogeneity of drug effects and the impact of the spatial microenvironment on drug actions.

SDMap provides user-friendly search and browsing interfaces for data access and visualization. On the “Search” page, users can input or select various types of keywords, such as the disease state (diseased or healthy) of tissue slices, tissue type, slice sample ID, drug name, or target (Fig. 2A). Additionally, the “Browse” page enables flexible access to data stored in the SDMap database (Fig. 2B), where users can filter desired data by clicking entries across different levels of the browsing tree—including drug name, disease state (diseased or healthy), and tissue type of slice. Through the “Search” and “Browse” pages, SDMap generates a data table where each row describes the effect of a specific drug-related instance on spots/cells within a spatial tissue slice. The descriptive information includes the following categories (Fig. 2C): (i) treatment instance details: drug name, cell line name (cell_iname), treatment dosage (pert_idose), and treatment duration (pert_itime); (ii) tissue slice information: tissue type (tissue) and slice sample ID (sample); (iii) spatial association metrics between treatment instances and cells across the tissue slice: promotion (or inhibition) spot ratio, defined as the proportion of spots on the tissue slice exhibiting a promoting (or inhibitory) effect in response to the drug-related instance; and (iv) additional details: basic information about the corresponding drug and tissue slice, along with the impact of the drug treatment instance on cell types and microenvironmental niches within the spatial context of the slice (Fig. 2D). On the results page, users can further filter entries via keyword searches. For each result entry, SDMap offers multi-feature interfaces to facilitate in-depth analysis.

#### Panel of easy-to-use analysis tools

Beyond data query and access, SDMap offers a suite of 10 easy-to-use tools for data retrieval, analysis, and visualization (Fig. 2E–M). Each tool executes a dedicated analytical pipeline by integrating diverse methodologies and multi-level data. In general, the input requirements for these tools include information related to the interesting tissue slice (e.g. disease status, tissue type, dataset, and sample ID) and drug-related treatment instance (such as drug name, treated cell context, dosage, and duration). However, the content displayed on the results page varies across tools. The Spatial Deconvolution tool enables exploration of the correlation between spatial cell identity and drug perturbation effects, as well as the perturbation effects of different drug concentrations, treatment durations, and cell contexts on the deconvolved spatial sites (Fig. 2E). The SVD (spatial variable drug) tool provides drugs with highly variable perturbation effects across spatial tissue slice under different treatment instances in terms of drug concentration, treatment durations, and cell contexts (Fig. 2F). Users can explore the spatial perturbation effects of drugs at different resolutions, including spot/cell, cluster, cell type, and microenvironment niche. Considering the drug action-related targets that affect the expression changes of downstream genes in the signaling network [32], therefore, exploring the association between the expression of spatial target genes and the drug perturbation effects can provide new insights into the mechanism of drug action. SDMap provides the SDT tool to dissect the spatial expression characteristics of target genes for drugs with known targets and dissect the association between spatial target activities and drug perturbation effects (Fig. 2G). The dynamic development of spatial microenvironmental heterogeneity is a driving factor that influences individual’s health status, drug effects, responses, and clinical outcomes [33, 34]. To characterize the spatial developmental landscape and identify driver features that impact spatial drug actions, SDMap constructs spatial developmental trajectory for each slice and offers Spatial Pseudotime tool to dissect the perturbation effects of drugs under different instance conditions along the pseudotime trajectory of spatial slice (Fig. 2H). Drug combinations, which enhance therapeutic efficacy while mitigating toxicity, hold significant value in the treatment of various complex diseases [35, 36]. Drug Combination tool can simulate the perturbation effects of combination drugs across spatial locations, and enables users to explore the combinatorial perturbation effects of drugs under two distinct instance backgrounds on different spots/cells, clusters, cell types, and niches within spatial tissue slice, as well as the spatial heterogeneity of these combinatorial effects (Fig. 2I). Exploring the dynamic changes of drug effects in tissue space is crucial for understanding the mechanisms of drug action. SDMap provides 3D reconstruction tool that leverages the SPACEL algorithm [30] to reconstruct 3D maps of tissue slices from the same patient and allows users to investigate the spatial heterogeneity of drug effects in three-dimensional space (Fig. 2J). As further supplements, the Dynamic 3D menu includes Dosage 3D and Time 3D tools, which can simulate the dynamic changes of the drug perturbation effects on cells on the spatially slices as the action time or dose changes. Dosage 3D (Time 3D) tool constructs a 3D model depicting the spatial impact of varying drug dosages (time of duration) on the same tissue slice under the same treatment duration (dosage) (Fig. 2K). In contrast to the 3D reconstruction function—which focuses on algorithm-driven reconstruction of multiple slices into a 3D structure—this tool centers on the associative visualization of drug dosage (treatment duration) gradients and spatial transcriptomic features under consistent treatment duration (dosage). Users can more intuitively observe how drug dosage (treatment duration) variations impact tissue cells at the spatial level, clearly capture the progressive trends and key differential regions of transcriptomic spatial features across the low-to-high dosage (treatment duration) spectrum, and obtain a robust visual analysis tool to dissect the spatial specificity of drug dosage (treatment duration) effects. The functional status of cells in the tumor microenvironment and their heterogeneity influence the efficacy of drug action and the variability in therapeutic responses [3, 37]. The Spatial Drug-CellStat tool enables users to explore the characteristic spatial changes in the activity of cancer-related functional states, hallmarks, and pathways in cancer tissue slices, as well as their associations with drug perturbations (Fig. 2L). It provides critical data support for revealing the spatial-specific mechanisms underlying cancer drug responses. The Spatial Single-cell tool offers spatial heterogeneity analysis of drug actions at spatial single-cell resolution (Fig. 2M). This tool provides the spatial distribution of cell clusters, cell types, and niches, as well as the perturbing effects of drug-specific instances (combinations of drug, duration time, dosage, and cellular context) on spatial cells. Moreover, SDMap also offers a “Help” page, which serves as a user guide presenting detailed descriptions of these tools and explanations for the results they produce.

### Example application

#### SDMap uncovers potential mechanisms of drug action and spatial heterogeneity

**Figure 3.**
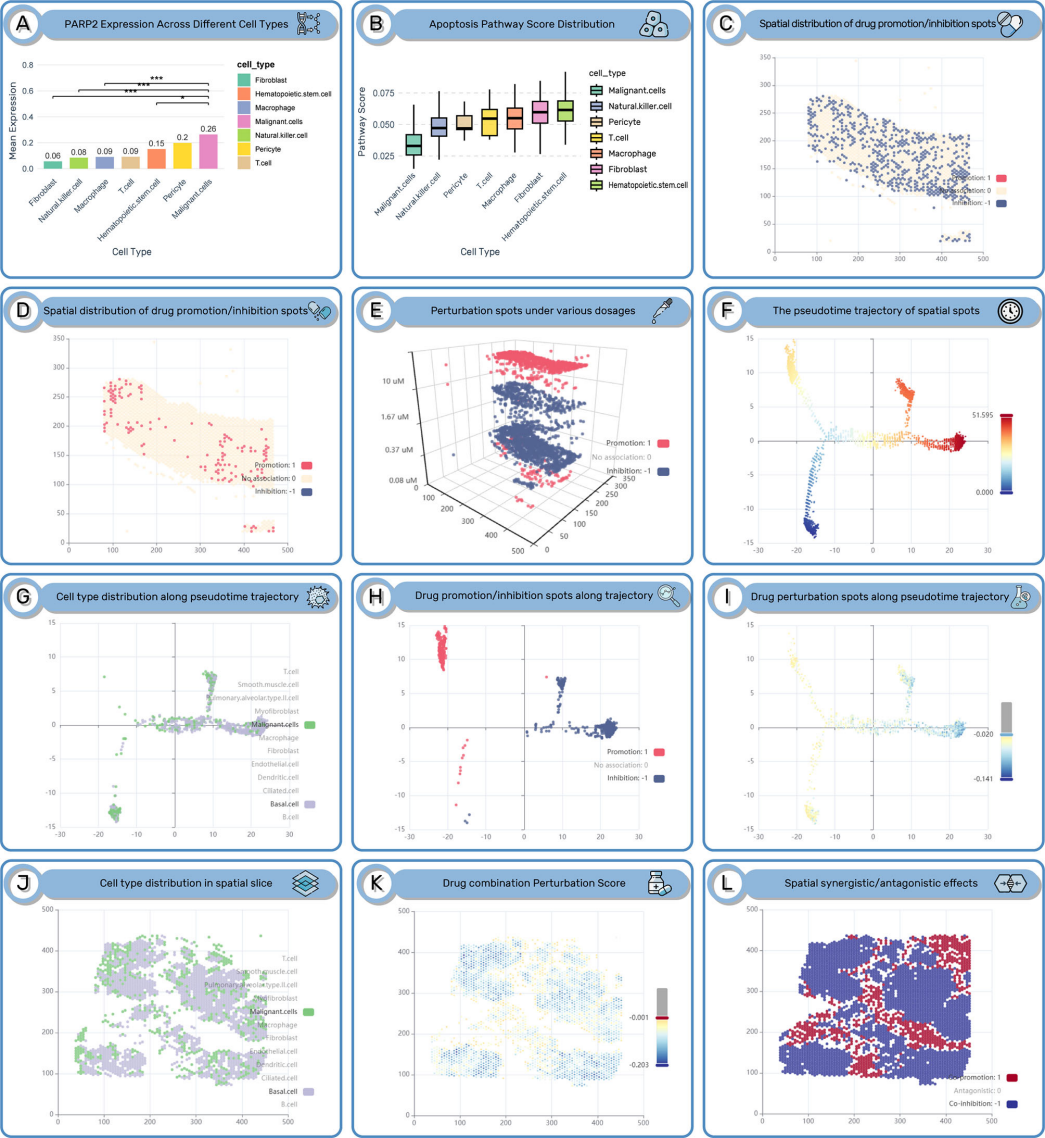
(**A**) The average expression levels of PARP2 across different spatial cell type regions of the slice (CytAssist_Fresh_Frozen_Human_Breast_Cancer). The Wilcoxon rank-sum test was used to calculate the difference in the expression of the PARP2 gene between spatial spots related with malignant cell types and other cell types (*P< .05, ***P< .001). (**B**) The activity of the apoptosis function in different spatial cell-type regions of the slice. (**C**) The perturbation effects of olaparib-related instance (dose: 0.12 µM, duration: 48 h, cell context: MCF7) on spots in the breast cancer slice are mainly inhibitory. (**D**) Results of the perturbation effects of olaparib (dose: 0.12 µM, duration: 6 h, cell context: MCF7) on spatial spots in the slice (mainly promotive), obtained using the SDT tool. (**E**) 3D plot of the perturbation effects of olaparib at different doses on spots in the slice. (**F**) Pseudotime trajectory of spots in the slice (CytAssist_FFPE_Human_Lung_Squamous_Cell_Carcinoma). (**G**) Distribution of malignant and basal spots on the pseudotime trajectory. (**H**) Distribution of spots inhibited (blue) and promoted (red) by the ceritinib-related instance (dose: 10 µM, duration: 24 h, cell context: NCI-H1437) on the pseudotime trajectory. (**I**) Distribution and perturbation effect scores of spots with inhibitory tendency (IS < 0) on the pseudotime trajectory of the slice (CytAssist_FFPE_Human_Lung_Squamous_Cell_Carcinoma) upon treatment with the panobinostat-related instance (dose: 1.11 µM, duration: 24 h, cell context: NCI-H1437). (**J**) Distribution of spots associated with malignant and basal cell types in the slice. (**K**) Combined perturbation effect scores of the two instances (the displayed spots have combined perturbation scores <0). (**L**) Combined perturbation effects of the two drugs on slice (red/blue: synergistic promotive/inhibitory effect).

Olaparib is a PARP inhibitor, mainly used for the treatment of breast cancer patients with specific gene mutations (such as BRCA1/2) [38]. However, the therapeutic effectiveness of this drug is still subject to certain limitations in clinical practice. To demonstrate the potential application of SDMap, we analyzed the effect of olaparib based on spatial tissue slices of breast cancer. On the “Search” page, input “Diseased-cancer” and “Breast” in the “Tissue” option, select “CytAssist_Fresh_Frozen_Human_Breast_Cancer” for “Slice,” and input “olaparib” as the drug name. According to the returned results, olaparib exerts a relatively strong inhibitory effect (inhibiting 35% of the spots) on breast cancer tissue under the action instance: dosage: 0.12 μM, time of duration: 48 h, and cell context: MCF7 (Supplementary Fig. S1). Further, click the SDT tool link corresponding to this entry to analyze the spatial expression of olaparib’s target and its perturbation effect on spatial spots. The results show that PARP2 is highly expressed in malignant spots on the spatial tissue section (Supplementary Fig. S2). Then, we used Wilcoxon rank-sum test to compare the significant differences in the expression of the target gene PARP2 of olaparib between malignant spot and other cell type-related spots (Fig. 3A). Functional annotation analysis in the KEGG database [39] showed that PARP2 is involved in the apoptosis function, and proteins encoded by upstream genes inhibit PARP2 to promote the apoptosis (Supplementary Fig. S3). As a PARP inhibitor, olaparib can promote tumor cell apoptosis by inhibiting PARP2. Using the Spatial Drug-CellStat tool for analysis, input “Functional Status” and “Apoptosis” in the “Functional dataset” and “Function” options, respectively. The returned results showed that the “apoptosis” function activity in the malignant spot regions of this slice is the lowest (Fig. 3B and Supplementary Fig. S4). The above results indicate that SDMap can help researchers reveal the relevant mechanisms of drug action and, to a certain extent, verify the reliability of the annotation of cell types in spatial regions.

On the result page of the SDT and Spatial Drug-CellStat tools, it was simultaneously found that the perturbation effect of olaparib on spatial cells of breast cancer slice (*CytAssist_Fresh_Frozen_Human_Breast_Cancer*) under the treatment instance (0.12 μM, 48 h, MCF7) is mainly inhibitory (Fig. 3C and Supplementary Fig. S5). Further analysis based on the results revealed that malignant spots account for 55% of the spatial spots inhibited by olaparib (321/583, Supplementary Fig. S6), but these inhibited malignant spots account for a limited proportion of all spatial malignant spots (321/1074). Analysis of the perturbation association between this instance and spatial niches showed that the enrichment degree of spatial spots inhibited by olaparib-related instance varies among different spatial niches (Supplementary Fig. S7). The above results indicate that the perturbation effect of drugs has spatial heterogeneity.

#### SDMap reveals that the perturbation effect of drugs is characterized by duration dependence and dose dependence

Next, we used SDMap to analyze the perturbation effects of drugs on the spatial microenvironment under different administration durations and dosages. Taking the analysis of olaparib’s perturbation effect on the spatial microenvironment in breast cancer tissue slice (*CytAssist_Fresh_Frozen_Human_Breast_Cancer*) as an example. First, under the same concentration (0.12 μM), we compared the spatial perturbation effects on spots in breast cancer tissue slice (*CytAssist_Fresh_Frozen_Human_Breast_Cancer*) under different administration durations (6 h, 24 h, and 48 h). The results showed that the perturbation effects of olaparib on the cells in the slice under 6 h, 24 h, and 48 h conditions were mainly promotion, no association, and inhibition, respectively (Fig. 3C and D and Supplementary Fig. S8). Further exploration of the differences in perturbation associations under different administration durations revealed that olaparib mainly exhibited a promoting effect on non-malignant spots in the spatial microenvironment under the 6 h condition (the proportion of promoted non-malignant spots was 110/137, while the proportion of promoted malignant spots was only 27/137, Supplementary Fig. S9). In contrast, under the 48 h condition, the proportion of inhibited malignant spots in the spatial microenvironment increased significantly (the proportion of inhibited malignant spots was 321/583, Supplementary Fig. S6). This indicates that under a certain concentration, the effect of olaparib on the spatial microenvironment may dynamically change from promoting immune cells in the microenvironment to inhibiting malignant cells in the microenvironment as the administration duration extends.

Then, we used SDMap to analyze and compare the spatial perturbation effects on cells in breast cancer tissue slice (*CytAssist_Fresh_Frozen_Human_Breast_Cancer*) under different dosages with the same administration duration. Using the Dosage 3D tool, the corresponding input parameters were selected as Diseased, Cancer, Breast, Breast Cancer, 10x Genomics, *CytAssist_Fresh_Frozen_Human_Breast_Cancer*, MCF7, olaparib, and 6 h. The returned results showed that, under the same administration duration (6 h), the perturbation effects of the drug on cells in the spatial microenvironment also changed dynamically with the increase of dosage, from the promoting effect on immune cells in the spatial microenvironment at low dosage → inhibitory effect on malignant cells → promoting effect on malignant cells (Fig. 3E and Supplementary Fig. S10). The occurrence of the promoting effect on malignant cells may be due to the faster development of drug resistance in malignant cells in the spatial microenvironment under the same administration duration as the dosage has reached a sufficiently high level [40].

The above results, on the one hand, reveal the spatial heterogeneity of drug effects, and on the other hand, indicate that the effective dosage and administration duration of drugs need to be precisely controlled during disease treatment, which is also one of the important contents in precision medicine research.

#### SDMap reveals potential drug combination for lung cancer

Ceritinib is a first-line clinical drug for the treatment of non-small cell lung cancer (NSCLC), but its effectiveness in some patients remains unclear [41]. Drug combination is an effective strategy to improve cancer treatment efficacy and reduce the emergence of drug resistance [42]. Here, we attempt to use SDMap to explore potential combination drugs with ceritinib for the treatment of NSCLC.

First, the SVD tool was used to retrieve drug-related instances with highly variable perturbation effects on the spatial tissue microenvironment of lung cancer. On the SVD page, the slice from the 10x Genomics dataset *CytAssist_FFPE_Human_Lung_Squamous_Cell_Carcinoma* was selected. In the returned results, the instance of ceritinib at 10 µM for 24 h (targeting the NSCLC cell context: NCI-H1437) showed variable spatial perturbation effects (Supplementary Fig. S11). Clicking on the ‘Details’ link corresponding to this instance, the visualization results revealed that ceritinib mainly inhibits malignant cells and basal cells in this slice (Supplementary Fig. S12). A recent study confirmed that abnormal basal cell clonal dynamics shape the development of early lung cancer [43]. The Spatial Pseudotime tool was used to analyze the perturbation effect of this instance on the temporal trajectory of malignant spots in the spatial microenvironment of NSCLC. Input parameters for Spatial Pseudotime tool: Diseased, Cancer, Lung, Lung Squamous Cell Carcinoma, CytAssist_FFPE_Human_Lung_Squamous_Cell_Carcinoma, NCIH1437, ceritinib,10 µM, and 24 h. In the result page, it was found that this ceritinib-related instance tends to inhibit basal-like cells and malignant cells in the late stage of the temporal trajectory (Fig. 3F–H and Supplementary Fig. S13), which is consistent with the clinical application of ceritinib as a first-line treatment for advanced NSCLC [41]. Further exploration using Spatial Pseudotime tool to identify drugs with potential combination effects with ceritinib revealed that the instance of panobinostat at 1.11 µM for 24 h (targeting the NCI-H1437 cell line) exerted a certain inhibitory effect on malignant cells in the early stage of the trajectory Fig. 3F and I), which could compensate for ceritinib’s insufficient inhibition of early stage malignant cells and basal-like cells (Fig. 3F–I). Analysis using the Spatial Deconvolution tool further confirmed that the inhibitory effects of both drugs increased with the increasing proportion of basal-like cells and malignant cells at spatial spots (Supplementary Fig. S14). In addition, the Drug Combination tool was used to analyze the combined effect of ceritinib and panobinostat (ceritinib-related instance: 10 µM, 24 h, and NCIH1437; panobinostat-related instance: 1.11 µM, 24 h, and NCIH1437) on slice CytAssist_FFPE_Human_Lung_Squamous_Cell_Carcinoma, which showed synergistic inhibitory effects on most basal-like and malignant spots (Fig. 3J–L). The above analysis results indicate that ceritinib and panobinostat have the potential for combined treatment of NSCLC, providing a reference for subsequent clinical combination strategies.

## Discussion

Characterizing the spatial distributions of drug-relevant cells is pivotal for elucidating the mechanisms underlying drug action and drug response. Currently, researchers have constructed a series of ST data resources, including STOmicsDB [10], SORC [11], CROST [12], and SODB [14]. Additionally, we built SPathDB, a comprehensive database for exploring spatial functional activity heterogeneity [13]. These databases provide valuable data resources for investigating the relationship between spatial microenvironment heterogeneity and disease pathological mechanisms, individual survival prognosis, and drug therapeutic responses. However, there is currently a lack of a comprehensive database that integrates spatial omics and pharmacogenomics data resources and is dedicated to exploring the effects of drugs in spatial contexts. Thus, we present SDMap, a comprehensive database that integrates large-scale ST and drug perturbation data while establishing associations between drug perturbations and cells/spots within spatial tissue contexts. SDMap encompasses associations across 5 490 079 spatial locations derived from 989 human tissue sections, along with 538 419 treatment instances corresponding to perturbations induced by 33 149 distinct drugs. SDMap also provides ten flexible analytical tools for exploring the perturbation effects of drugs on the spatial microenvironment under specific dosage and duration conditions, as well as the spatial heterogeneity of drug actions. Meanwhile, case studies have confirmed the applications of SDMap in revealing mechanisms related to drug actions and exploring the potential of new combination therapies. Notably, SDMap can provide the perturbation effects of drugs on the spatial microenvironment under specific experimental contexts (such as dosages, durations, and cell contexts), which is of more practical significance for guiding subsequent further research. SDMap can serve as a powerful complement to current data resources related to ST and pharmacogenomics. The SDMap database can provide strong support for research on drug action mechanisms, spatial cell-targeted therapies, the discovery of new spatial therapeutic targets, and the development of therapeutic strategies. We will continue to update the SDMap database in the following aspects: (i) continuous collection of spatial omics data and drug perturbation data. Currently, drug perturbation data are primarily derived from cell lines; we will consider integrating the data related to tissue or clinical samples in the future; (ii) given the limited number of currently integrated single-cell resolution datasets, further expanding single-cell resolution spatial omics data in the future; and (iii) further improving downstream analysis tools (e.g. the “Drug Combination” tool only supports the spatial combined effect analysis of two drugs; we will consider enabling the spatial combined effect analysis of multiple drugs in future updates).

## Supplementary Material

gkaf1046_Supplemental_File

## Data Availability

SDMap is freely available online at http://bio-bigdata.hrbmu.edu.cn/SDMap/.
