# Supplementary material for: SDMap: a comprehensive database of spatial drug perturbation maps
Source: Nucleic Acids Res. 2025 Oct 22;54(D1):D1634–45. doi: 10.1093/nar/gkaf1046 (PMC12807743; doi:10.1093/nar/gkaf1046)
Supplement: gkaf1046_Supplemental_File [file gkaf1046_supplemental_file.pdf]

Supplementary Methods

Cell annotation of scRNA-seq data for spatial spot deconvolution

We combined two automated methods, scMayoMap [1] and SingleR (https://doi.org/doi:10.18129/B9.bioc.SingleR), with a manual annotation strategy to annotate cell types. For each scRNA-seq dataset, the detailed steps are as follows: (i) perform automatic annotation using scMayoMap [1] and SingleR (https://doi.org/doi:10.18129/B9.bioc.SingleR). ScMayoMap accurately identifies and annotates cell types in different tissue contexts. We use the standard clustering results from upstream analyses as input, select specific tissue types, and annotate each cluster with the corresponding cell type based on the cell markers in scMayoMap [1]. For SingleR, we extract the expression matrix using the 'GetAssayData' function and select appropriate reference datasets and annotated cell types based on known cell type labels from reference datasets using the 'SingleR' function. The consistency of the two automatic annotation results is manually reviewed, and the consistent annotations from both methods are adopted. For clusters with inconsistent annotation results, we collect cell type marker genes from the CellMarker 2.0 database [2] and combine these marker genes with the feature genes obtained from upstream analyses to manually correct the automatic annotation results. For a small number of datasets without corresponding tissue types in scMayoMap, we only use SingleR for automatic annotation, followed by further manual review and correction based on manual annotation. For cancer-related scRNA-seq datasets, the inferCNV method (https://github.com/broadinstitute/infercnv) is further used to identify malignant cell types. All immune cells in each dataset are

used as reference normal cells. We calculated the inferred copy number variation (CNV) scores of cells [3]. Then, we compared the inferred CNV scores of cells in each cluster with those of reference cells. Cell clusters with significantly higher inferred CNV scores compared to reference cells (Wilcoxon rank-sum test P-value < 0.05) are defined as malignant cells.

Determination of perturbation associations between drug-related instances and spatial spots

Constructing null distribution of the perturbation effect score (IS). Further, the significance of the perturbation effect of drug-related instances on spatial spots was evaluated. SDMap constructs a null distribution of the perturbation effect score (IS) of drug-related instances on spatial spots, and the specific steps are as follows:

(i) Respectively, 100,000 upregulated drug perturbation gene sets and 100,000 downregulated drug perturbation gene sets with the same distribution of gene sets were randomly simulated according to the number of genes in the real instance perturbation-related upregulated and downregulated signature gene sets;

(ii) For each slice, 20% of the spatial spots were randomly selected, and the AUCell algorithm was used to calculate the IS scores of the simulated upregulated and downregulated perturbation gene sets in (i);

(iii) For each randomly selected spot/cell, the difference between the AUCScore of the simulated upregulated gene set and that of the simulated downregulated gene set (i.e., IS score) was calculated, which was taken as the background IS score of the null distribution.

Determination of drug instance-spatial spot perturbation association. Then, the significance thresholds were determined based on the constructed null distribution of IS scores: all background scores were sorted, and the scores corresponding to the top 2.5% (bottom 2.5%) of the sorted results were taken as the thresholds for significant promotion (significant inhibition) of spatial spots by drug-related instances. Finally, a binary matrix of perturbation effects between drug-related instances and spatial spots was constructed according to the determined thresholds. For values in the instance-spatial spot perturbation effect IS scoring matrix that are greater than (less than) the significant promotion (inhibition) threshold, they are defined as the instance having a significant promotion (inhibition) effect on the corresponding spatial spot, and assigned a value of 1 (-1); the remaining values in the matrix are assigned a value of 0, indicating that the corresponding instance has no significant perturbation effect on the spatial spot.

In addition, for spatial tissue slice datasets with spot resolution and single-cell resolution, null distributions of perturbation effect scores (IS) were constructed respectively to determine the significance of perturbation effects and perturbation associations between drug-related instances and spatial spots or cells. For spot resolution datasets: the threshold for significant promotion (top 2.5%) is 0.1105, and the threshold for significant inhibition (bottom 2.5%) is -0.1353. For cell resolution datasets, the corresponding threshold for significant promotion (inhibition) is 0.7917 (-0.8612).

Non-negative transformation of perturbation effect score (IS)

For each spatial slice, the perturbation effect score (IS) is non-negative transformed as follows:

$$IS_{i,j}^{t} = \frac{IS_{i,j} - IS_{min}}{IS_{max} - IS_{min}}$$

$$IS_{i,j}' = e^{IS_{i,j}^{t}}$$

Here, IS_{max} and IS_{min} represent the maximum and minimum values on the IS profile for slice, respectively; $IS_{i,j}$ denotes the perturbation effect score of drug i in spot j; and $IS_{i,j}'$ refers to the non-negative transformed value of drug i in spot j.

References of Supplementary Methods

1. Yang, L., Ng, Y.E., Sun, H., Li, Y., Chini, L.C.S., LeBrasseur, N.K., Chen, J. and Zhang, X. (2023) Single-cell Mayo Map (scMayoMap): an easy-to-use tool for cell type annotation in single-cell RNA-sequencing data analysis. *BMC Biol*, **21**, 223.
2. Hu, C., Li, T., Xu, Y., Zhang, X., Li, F., Bai, J., Chen, J., Jiang, W., Yang, K., Ou, Q. *et al.* (2023) CellMarker 2.0: an updated database of manually curated cell markers in human/mouse and web tools based on scRNA-seq data. *Nucleic Acids Res*, **51**, D870-D876.
3. Sun, K., Xu, R., Ma, F., Yang, N., Li, Y., Sun, X., Jin, P., Kang, W., Jia, L., Xiong, J. *et al.* (2022) scRNA-seq of gastric tumor shows complex intercellular interaction with an alternative T cell exhaustion trajectory. *Nat Commun*, **13**, 4943.

Supplementary Table

Table S1. ScRNA-seq datasets used for cell-type deconvolution of spot-resolution slices

Datasets_ID	Tissue	State	Disease_name	PMID
GSE115978	Skin	diseased	Melanoma	30388455
GSE129845	Bladder	diseased	Bladder Cancer	31462402
GSE132465	Colon	diseased	Colorectal Cancer	32451460
GSE139448	Brain	diseased	Glioblastoma	32004492
GSE140819	Lung	diseased	non-small cell lung carcinoma	32405060
GSE151530	Liver	diseased	Hepatocellular carcinoma	34216724
GSE152938	Kidney	diseased	Re-l Cell Carcinoma	34722263
GSE154600	Ovary	diseased	High-grade serous ovarian carcinoma	-
GSE165399	Pancreas	diseased	Intraductal papillary mucinous neoplasm,Pancreatic adenosquamous carcinoma	34326696
GSE168652	cervical	diseased	Cervical Cancer	-
GSE180286	Breast	diseased	Breast Cancer	34611125
GSE184198	Gastric	diseased	Gastric Cancer	36372898
GSE185344	Prostate	diseased	Prostate Cancer	36229464
GSE188711	Colon	diseased	Colorectal Cancer	34793335
GSE193960	Lung	diseased	Small Cell Lung Cancer	-
GSE195832	Head And Neck Oral	diseased	Head And Neck Oral Cavity Squamous Cell Carcinoma	35262677
GSE202371	Brain	diseased	Gliomas	36336787
GSE203612	Breast	diseased	Breast Cancer	35931863
GSE203612	Endometrium	diseased	Endometrial Carcinoma	35931863
GSE203612	Stomach	diseased	Gastric Stromal Tumor	35931863
GSE203612	Liver	diseased	hepatocellular carcinoma	35931863
GSE203612	Ovary	diseased	Ovarian Cancer	35931863
GSE211956	Ovary	diseased	Ovarian Cancer	38570491
GSE215403	Oral	diseased	Oral Squamous Cell Carcinoma	37792582
GSE218170	Skin	diseased	Cutaneous Squamous Cell Carcinoma	37669956
GSE224333	Ovary	diseased	Ovarian Clear Cell Carcinoma	38670097
GSE228501	Testis	diseased	Testicular Germ Cell Tumor	38123589
GSE235931	Ovary	diseased	High Grade Serous Ovarian Carcinoma	38328306
GSE236698	Colon	diseased	Mucinous Colorectal Adenocarcinoma	38778448
GSE250521	Thyroid	diseased	Thyroid Cancer	-
GSE261917	Lymph Node	diseased	Lymphoma	-
GSE261957	Liver	diseased	Hepatoblastoma	39900120
GSE263521	Peritoneum	diseased	Desmoplastic Small Round Cell Tumor	38781959
GSE270667	Lung	diseased	Lung Cancer	-

GSE156405	Pancreas	diseased	pancreatic cancer	34426439
GSE179373	brain	diseased	lung squamous cell carcinoma	35584630
GSE200218	Skin	diseased	Melanoma	35803246
GSE140042	Head And Neck	diseased	head and neck squamous cell carcinoma	
GSE150660	brain	diseased	human leptomeningeal metastasis	32675368
GSE281288	pancreas	diseased	pancreatic cancer	39827463
GSE219121	Spi-l Cord	GSE228 360	-	37095395
GSE228360	Ileal	GSE228 360	-	40059828
GSE129308	Brain	healthy	-	-
GSE134355	Brain	healthy	-	32214235
GSE134355	Cerebell um	healthy	-	32214235
GSE134355	Kidney	healthy	-	32214235
GSE134355	Liver	healthy	-	32214235
GSE134355	Lung	healthy	-	32214235
GSE153760	Skin	healthy	-	32344053
GSE176499	Colon	healthy	-	-
GSE185477	Liver	healthy	-	34792289
GSE190442	Spi-l Cord	healthy	-	36731429
GSE200161	Adipose	healthy	-	37474525
GSE211630	Oral	healthy	-	37555396
GSE212244	Thymus	healthy	-	40315299
GSE239890	Heart	healthy	-	-
GSE241132	Skin	healthy	-	39468077
GSE248609	Anterior tibial artery	healthy	-	39395983
GSE253200	Knee	healthy	-	39919907
GSE260658	Endomet rium	healthy	-	39471215
GSE260658	Myomet rium	healthy	-	39471215
GSE260685	Ovary	healthy	-	-
GSE280984	Pancreas	healthy	-	39763987
GSE282362	Muscle	healthy	-	39755918

Supplementary Figures

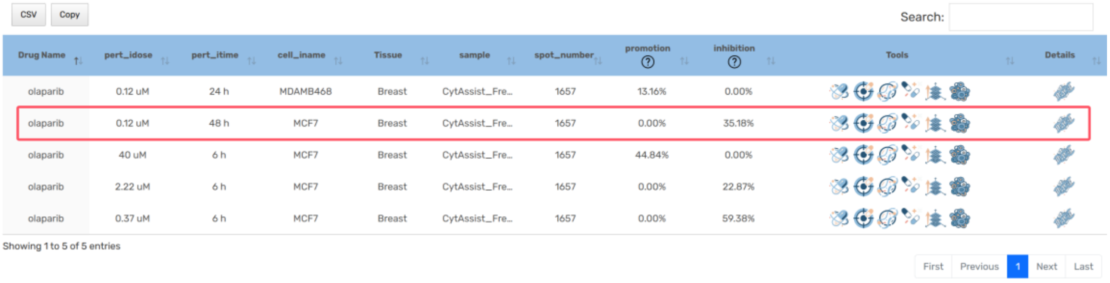

CSV Copy

Search:

Drug Name	pert_idose	pert_itime	cell_iname	Tissue	sample	spot_number	promotion	inhibition	Tools	Details
olaparib	0.12 uM	24 h	MDAMB468	Breast	CytAssist_Fre...	1657	13.16%	0.00%		
olaparib	0.12 uM	48 h	MCF7	Breast	CytAssist_Fre...	1657	0.00%	35.18%		
olaparib	40 uM	6 h	MCF7	Breast	CytAssist_Fre...	1657	44.84%	0.00%		
olaparib	2.22 uM	6 h	MCF7	Breast	CytAssist_Fre...	1657	0.00%	22.87%		
olaparib	0.37 uM	6 h	MCF7	Breast	CytAssist_Fre...	1657	0.00%	59.38%		

Showing 1 to 5 of 5 entries

First Previous 1 Next Last

Figure S1. The result page after entering Tissue: *Diseased-cancer -> Breast*, Slice: *CytAssist_Fresh_Frozen_Human_Breast_Cancer*, and Drug name: *olaparib* on the 'search' page.

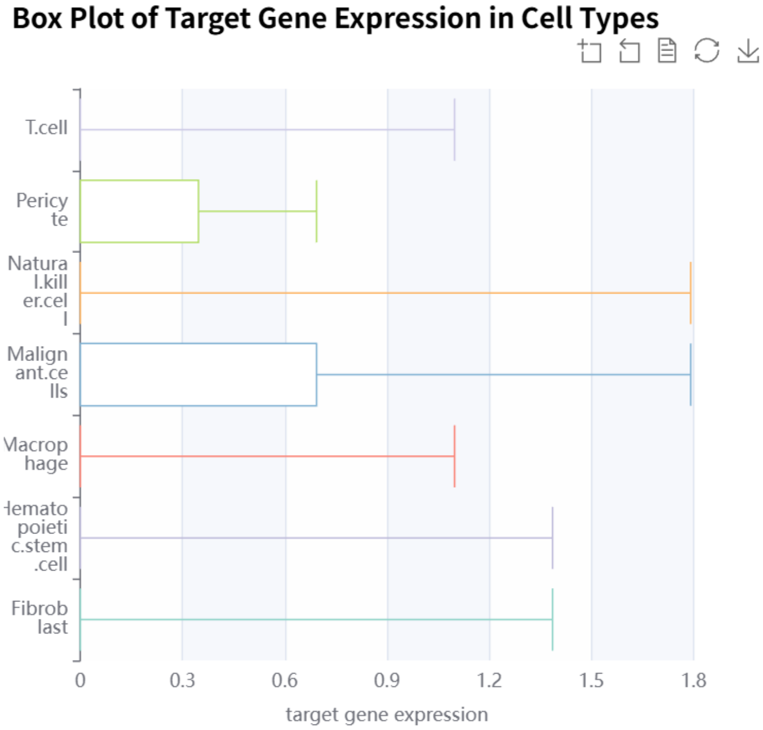

Figure S2. The expression levels of PARP2, the target of Olaparib obtained by the 'SDT' tool, in spots of different cell type regions within the slice (CytAssist_Fresh_Frozen_Human_Breast_Cancer) in spatial context.

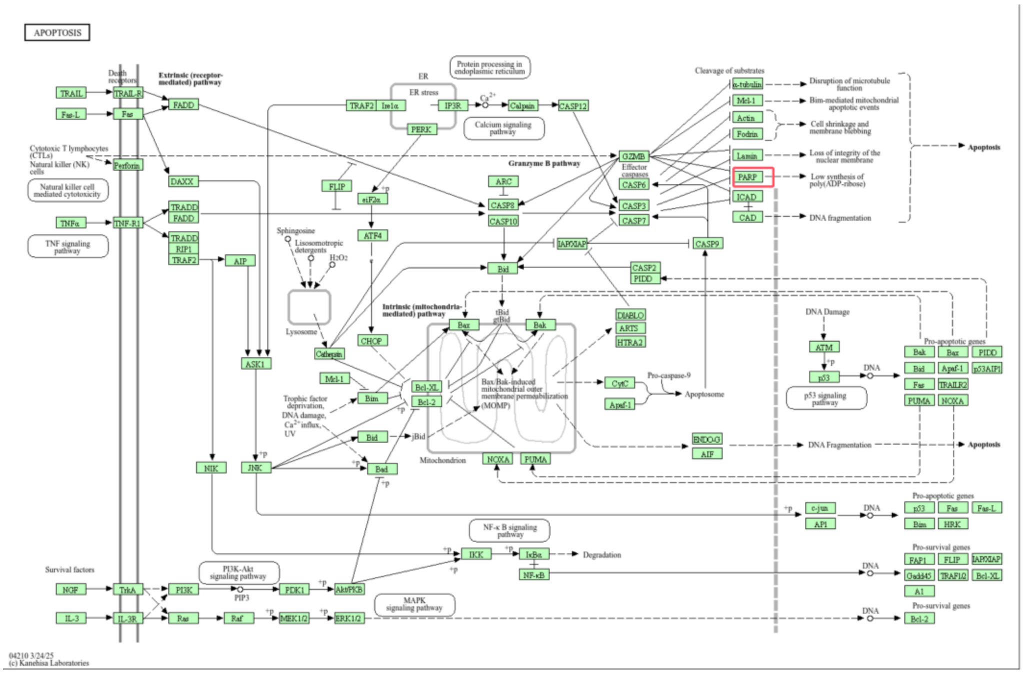

Figure S3. Annotation of PARP2, the target of Olaparib, in the Apoptosis (hsa04210) pathway from KEGG (with the node marked by a red box). The pathway map is derived from KEGG (https://www.kegg.jp/pathway/hsa04210).

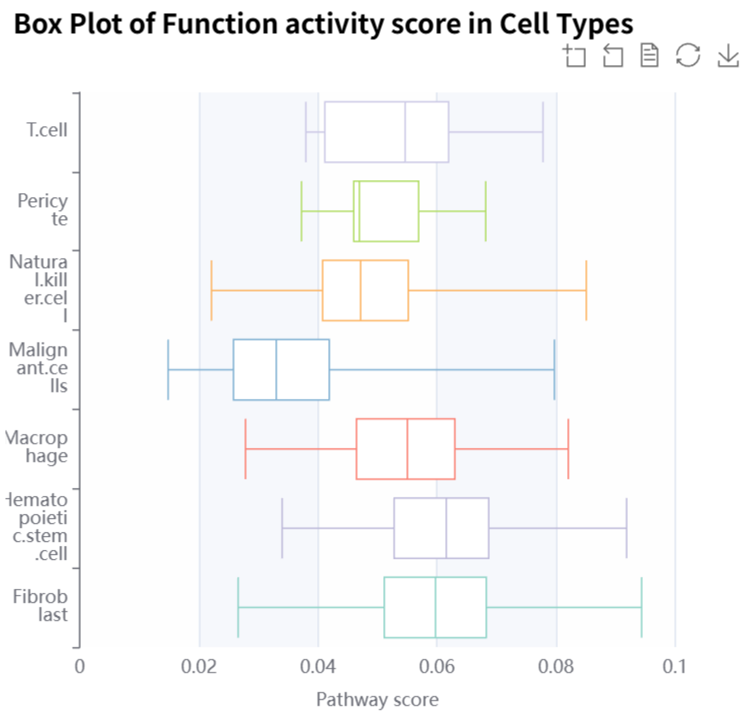

Figure S4. The activity of 'Apoptosis' function in different spatial cell type regions on the slice *CytAssist_Fresh_Frozen_Human_Breast_Cancer*, analyzed by the 'Spatial Drug-CellStat' tool. Detailed input parameters: Breast, Breast cancer, 10×Genomics, CytAssist_Fresh_Frozen_Human_Breast_Cancer, MCF7, olaparib, 0.12uM,48h, Functional Status and Apoptosis.

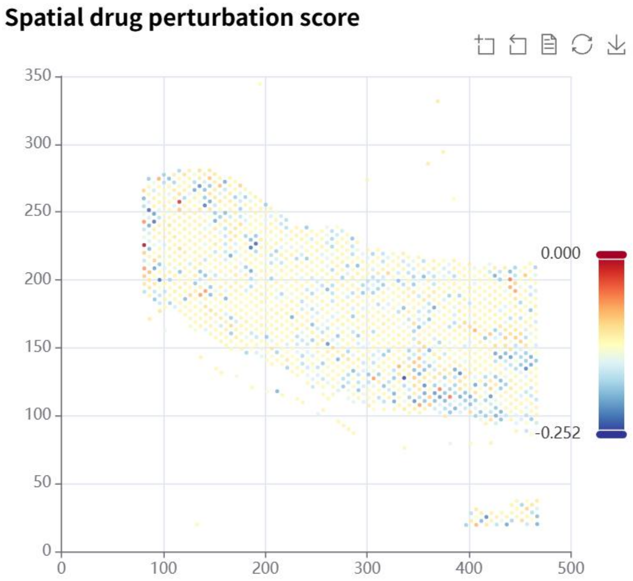

Figure S5. The perturbation effect score of Olaparib-related instances (for MCF7 cell line, drug dose 0.12uM, duration 48h) on spatial spots of the breast cancer slice (CytAssist_Fresh_Frozen_Human_Breast_Cancer), which can be obtained from the result page of ‘SDT’ and ‘Spatial Drug-CellStat’ tools.

Association Of Olaparib And Cell Types In CytAssist_Fresh_Frozen_Human_Breast_Cancer

celltype	cells_in_celltype	Celltype Neg/Total_Neg	p_negative	Celltype Pos/Total_Pos	p_positive	association
Fibroblast	89	39/583	5.20e-2	0/0	1	no association
Hematopoietic.st...	52	21/583	2.55e-1	0/0	1	no association
Macrophage	104	68/583	9.07e-11	0/0	1	inhibition
Malignant.cells	1074	321/583	1.00e+0	0/0	1	no association
Natural.killer.cell	319	127/583	3.20e-2	0/0	1	no association

Showing 1 to 5 of 7 entries

First Previous 1 2 Next Last

Figure S6. The distribution of promotion and inhibition spots across different cell type regions in the breast cancer spatial slice (Slice: *CytAssist_Fresh_Frozen_Human_Breast_Cancer*) caused by olaparib-related instances (acting on the MCF7 cell line at a concentration of 0.12uM for 48h). The numbers in the red color boxes respectively represent the number of malignant spots in this slice (1074), and the number of malignant spots inhibited by the instance / the total number of spots inhibited by the instance in this slice (321/583).

Association Of Olaparib And Niches In CytAssist_Fresh_Frozen_Human_Breast_Cancer

niches	cells_in_niche	Niche Neg/Total_Neg	p_negative	Niche Pos/Total_Pos	p_positive	association
1	265	75/583	9.96e-1	0/0	1	no association
2	83	39/583	1.53e-2	0/0	1	no association
3	103	52/583	7.14e-4	0/0	1	no association
4	25	17/583	7.79e-4	0/0	1	no association
5	45	26/583	1.42e-3	0/0	1	no association

Showing 1 to 5 of 12 entries

First Previous 1 2 3 Next Last

Figure S7. The distribution and enrichment significance of promotion and inhibiton spots in different spatial niches within the breast cancer spatial slice (Slice: *CytAssist_Fresh_Frozen_Human_Breast_Cancer*) induced by olaparib-related instance (acting on MCF7 cell context at a concentration of 0.12uM for 48h).

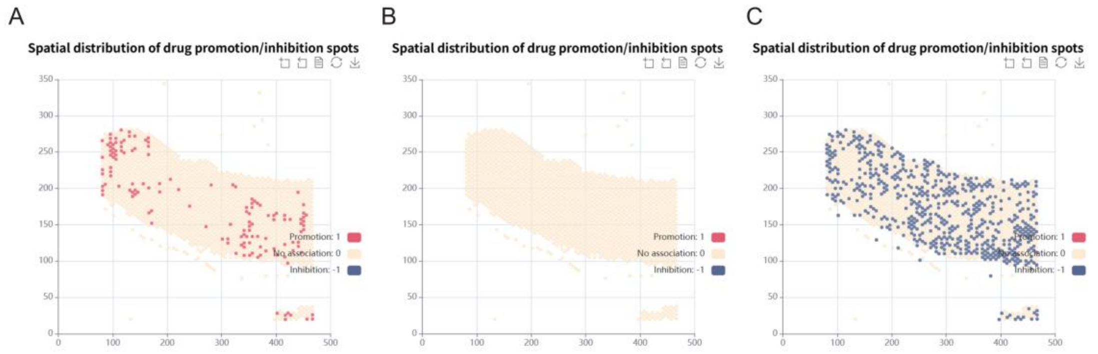

Figure S8. Analysis of the perturbation effects of olaparib on spatial spots at the same dose but different treatment durations using the 'SDT' tool. (A-C) Perturbation effects of three Olaparib instances (on MCF7 cell line, at a concentration of 0.12uM, with treatment durations of 6h, 24h, and 48h respectively) on spatial spots of the slice (*CytAssist_Fresh_Frozen_Human_Breast_Cancer*) from the 10×Genomics dataset. Blue spots: spots inhibited by the instance; red spots: spots promoted by the instance; yellow spots: spots with no significant association.

Association Of Olaparib And Cell Types In CytAssist_Fresh_Frozen_Human_Breast_Cancer

celltype	cells_in_celltype	Celltype Neg/Total_Neg	p_negative	Celltype Pos/Total_Pos	p_positive	association
Fibroblast	89	0/0	1	18/137	2.02e-4	promotion
Hematopoietic.st...	52	0/0	1	23/137	9.71e-13	promotion
Macrophage	104	0/0	1	20/137	1.81e-4	promotion
Malignant.cells	1074	0/0	1	27/137	1	no association
Natural.killer.cell	319	0/0	1	40/137	2.20e-3	no association

Showing 1 to 5 of 7 entries

First Previous 1 2 Next Last

Figure S9. The distribution of promotion and inhibiton spots across different cell type regions in the slice (*CytAssist_Fresh_Frozen_Human_Breast_Cancer*) from the 10×Genomics dataset, as obtained by the 'SDT' tool for Olaparib at a dose of 0.12uM with a treatment duration of 6h (cell context: MCF7). The numbers in the red box represent: the number of malignant spots promoted by the instance / the total number of spots promoted by the instance in this slice (27/137).

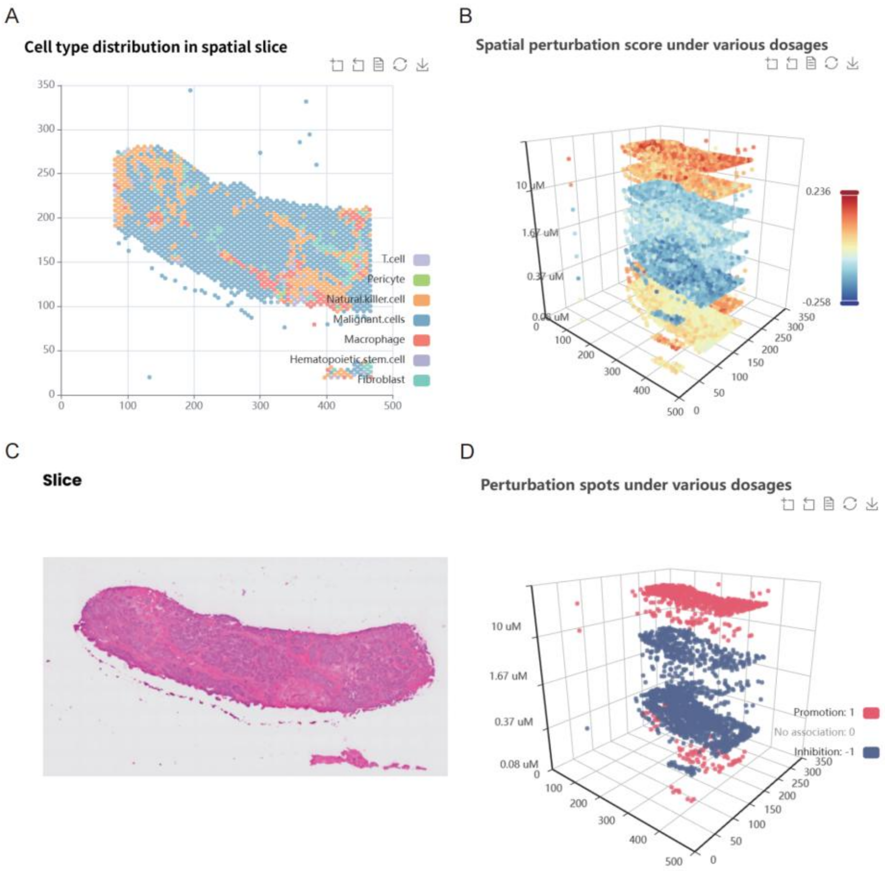

Figure S10. Analysis of the perturbation effects of different drug doses on the spatial microenvironment of breast cancer tissue slice (*CytAssist_Fresh_Frozen_Human_Breast_Cancer*) under the condition of the same treatment duration (6h) using the 'Dosage 3D' tool. The corresponding input parameters were selected as follows: Diseased, cancer, Breast, breast cancer, 10×Genomics, CytAssist_Fresh_Frozen_Human_Breast_Cancer, Olaparib and 6h. (A) Spatial distribution of cell types on the slice. (B) 3D plot of perturbation effects of Olaparib at different doses on spots in the slice (*CytAssist_Fresh_Frozen_Human_Breast_Cancer*) (with continuous values for IS score). (C) Histological image of the slice *(CytAssist_Fresh_Frozen_Human_Breast_Cancer*). (D) 3D plot of perturbation effects of Olaparib at different doses on spots in the slice (*CytAssist_Fresh_Frozen_Human_Breast_Cancer*). Blue spots: spots inhibited by Olaparib-related instance; red spots: spots promoted by Olaparib-related instance; yellow spots: spots with no significant association.

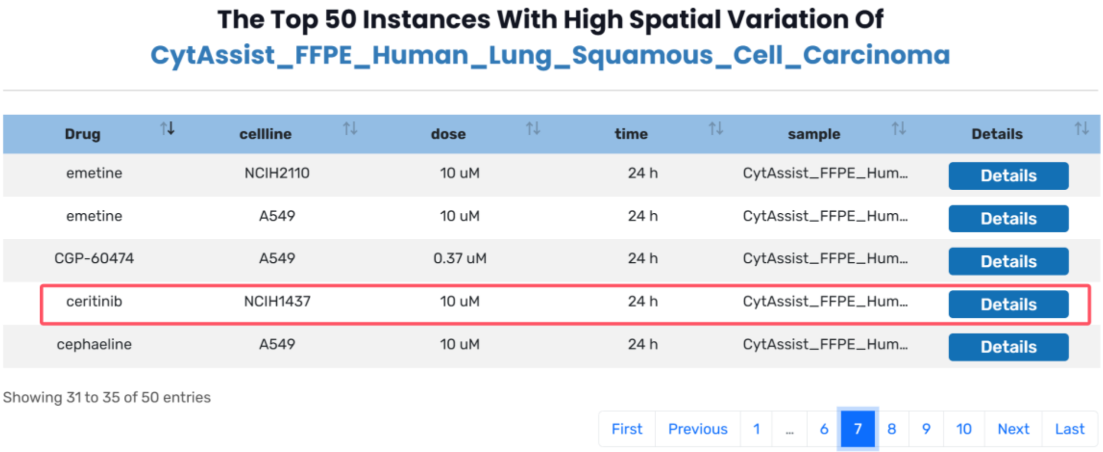

The Top 50 Instances With High Spatial Variation Of
CytAssist_FFPE_Human_Lung_Squamous_Cell_Carcinoma

Drug	cellline	dose	time	sample	Details
emetine	NCIH2110	10 uM	24 h	CytAssist_FFPE_Hum...	Details
emetine	A549	10 uM	24 h	CytAssist_FFPE_Hum...	Details
CGP-60474	A549	0.37 uM	24 h	CytAssist_FFPE_Hum...	Details
ceritinib	NCIH1437	10 uM	24 h	CytAssist_FFPE_Hum...	Details
cephaeline	A549	10 uM	24 h	CytAssist_FFPE_Hum...	Details

Showing 31 to 35 of 50 entries

First Previous 1 ... 6 7 8 9 10 Next Last

Figure S11. On the 'SVD' page, select the slice from the 10XGenomics dataset: *CytAssist_FFPE_Human_Lung_Squamous_Cell_Carcinoma*. In the list of instances with highly variable spatial perturbation effects on the returned result page, the instance of ceritinib at 10uM concentration for 24h (this instance targets the non-small cell lung cancer cell line NCIH1437) exhibits highly variable spatial perturbation effect (marked by the red box). The parameters input in the SVD interface are as follows: Diseased, cancer, Lung, Lung Squamous Cell Carcinoma, 10 × Genomics and CytAssist_FFPE_Human_Lung_Squamous_Cell_Carcinoma.

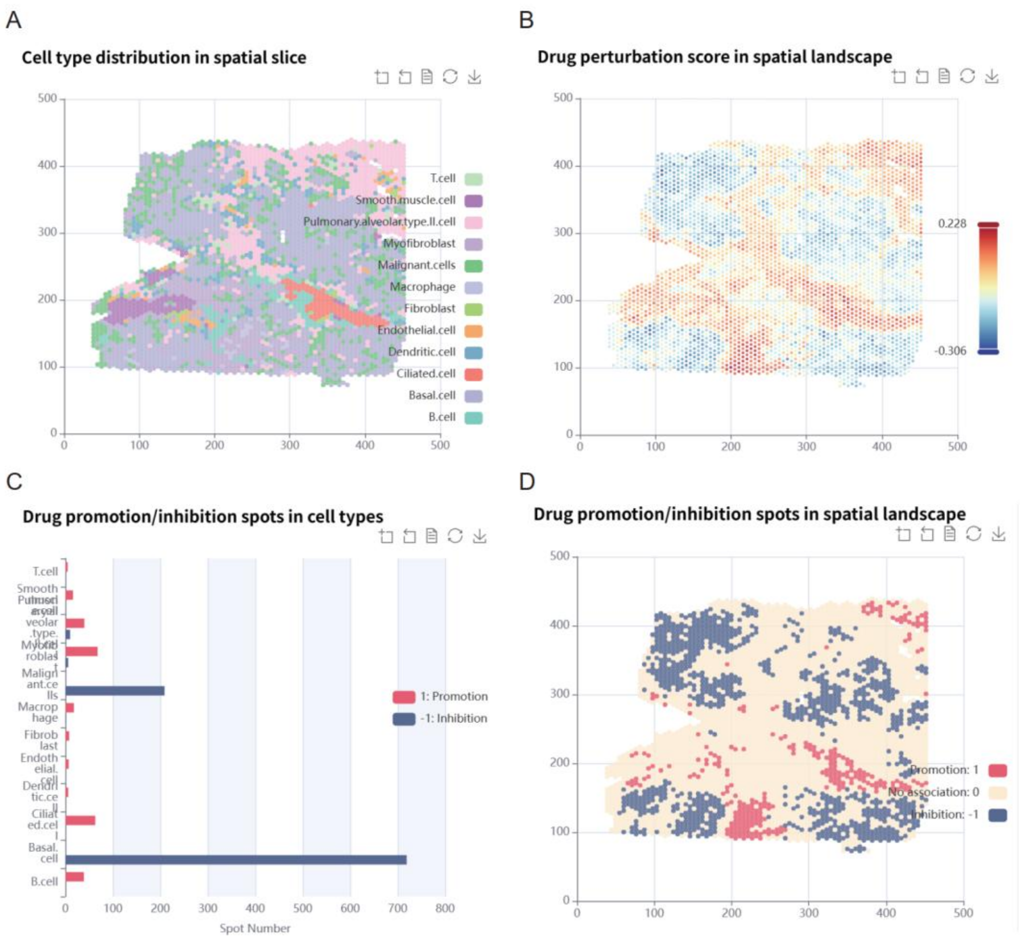

Figure S12. On the 'SVD' page, select the slice from the 10XGenomics dataset: *CytAssist_FFPE_Human_Lung_Squamous_Cell_Carcinoma*. Check the perturbation effect of the ceritinib instance (10uM for 24h) on spots in the spatial microenvironment on the returned result page. (A) Spatial distribution of cell types in the slice. (B) Perturbation scores of the ceritinib-related instance (dose: 10uM, Duration: 24h, cell context: NCI-H1437) on spatial spots. (C) Distribution of spots promoted (red) and inhibited (blue) by the ceritinib-related instance (dose: 10uM, Duration: 24h, cell context: NCI-H1437) across different spatial cell types. (D) Spatial distribution of spots promoted (red) and inhibited (blue) by the ceritinib-related instance (dose: 10uM, Duration: 24h, cell context: NCI-H1437).

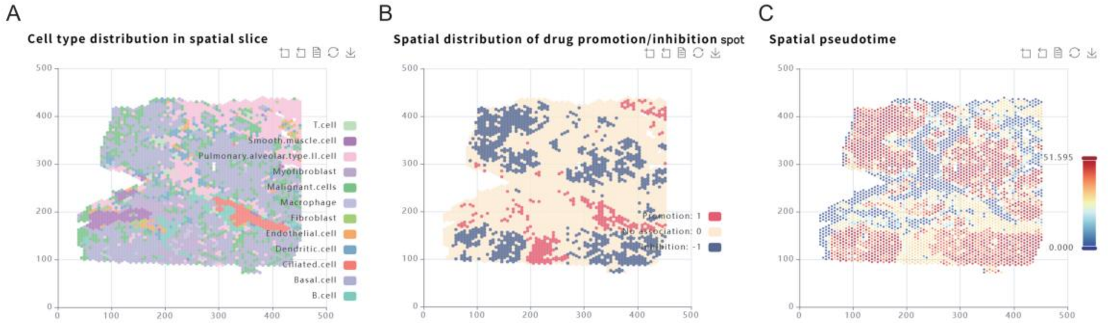

Figure S13. Analysis of the perturbation effect of the ceritinib-related instance (dose: 10uM, Duration: 24h, cell context: NCI-H1437) on the malignant and basal spots along the pseudotime trajectory of slice (CytAssist_FFPE_Human_Lung_Squamous_Cell_Carcinoma) using the Spatial Pseudotime tool. (A) Spatial distribution of cell types in the slice. (B) Spatial distribution of spots promoted (red) and inhibited (blue) by the ceritinib-related instance (dose: 10uM, Duration: 24h, cell context: NCI-H1437). (C) Pseudotime for spatial spots. Input parameters: Diseased, cancer, Lung, Lung Squamous Cell Carcinoma, CytAssist_FFPE_Human_Lung_Squamous_Cell_Carcinoma, NCIH1437, ceritinib,10uM and 24h.

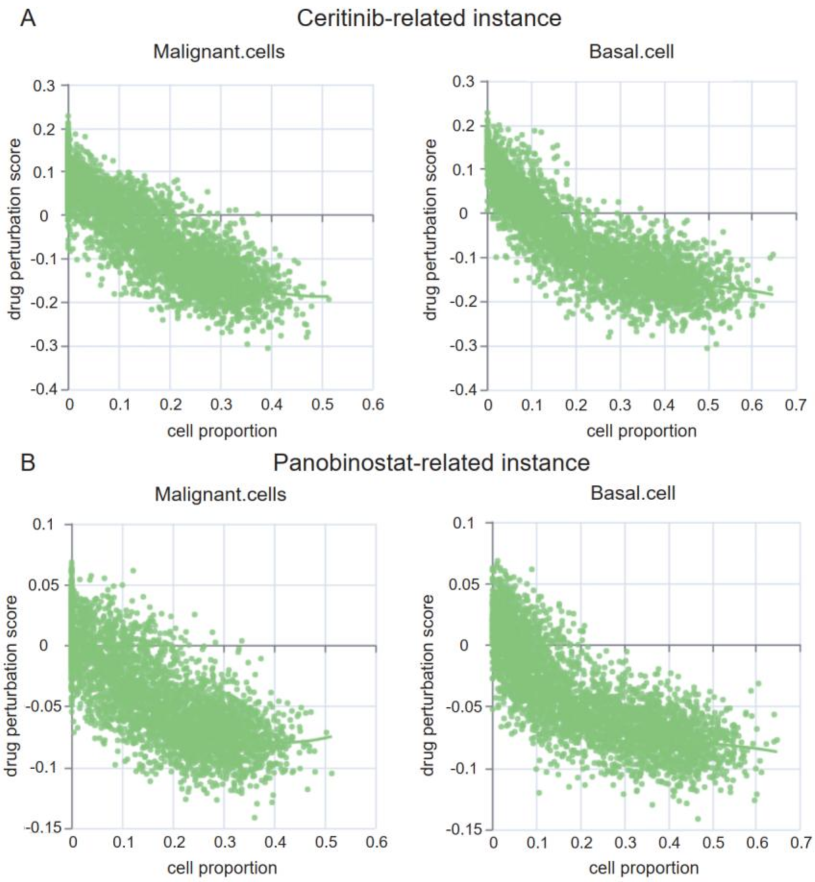

Figure S14. (A) Associations between the perturbation effects of the ceritinib-related instance (dose: 10uM, Duration: 24h, cell context: NCI-H1437) on spots in the spatial slice (CytAssist_FFPE_Human_Lung_Squamous_Cell_Carcinoma) and the proportions of malignant cells and basal cells at the spots. The results were obtained using the Spatial Deconvolution tool, with input parameters: Diseased, cancer, Lung, Lung Squamous Cell Carcinoma, CytAssist_FFPE_Human_Lung_Squamous_Cell_Carcinoma, NCIH1437, ceritinib,10uM and 24h. (B) Associations between the perturbation effects of the panobinostat-related instance (dose: 1.11uM, Duration: 24h, cell context: NCI-H1437) on spots in the spatial slice (CytAssist_FFPE_Human_Lung_Squamous_Cell_Carcinoma) and the proportions of malignant cells and basal cells at the spots. The results were obtained using the Spatial Deconvolution tool, with input parameters: Diseased, cancer, Lung, Lung Squamous Cell Carcinoma, CytAssist_FFPE_Human_Lung_Squamous_Cell_Carcinoma, NCIH1437, panobinostat,1.11uM and 24h.
